# Sparse-Coding-Based Computed Tomography Image Reconstruction

**DOI:** 10.1155/2013/145198

**Published:** 2013-02-26

**Authors:** Sang Min Yoon, Gang-Joon Yoon

**Affiliations:** ^1^School of Computer Science, Kookmin University, 77 Jeongneung-ro, Sungbuk-gu, Seoul 136-702, Republic of Korea; ^2^National Institute for Mathematical Science, KT Daeduk 2 Research Center, 463-1 Jeonmin-dong, Yuseong-gu, Daejeon 305-811, Republic of Korea

## Abstract

Computed tomography (CT) is a popular type of medical imaging that generates images of the internal structure of an object based on projection scans of the object from several angles. There are numerous methods to reconstruct the original shape of the target object from scans, but they are still dependent on the number of angles and iterations. To overcome the drawbacks of iterative reconstruction approaches like the algebraic reconstruction technique (ART), while the recovery is slightly impacted from a random noise (small amount of *ℓ*_2_ norm error) and projection scans (small amount of *ℓ*_1_ norm error) as well, we propose a medical image reconstruction methodology using the properties of sparse coding. It is a very powerful matrix factorization method which each pixel point is represented as a linear combination of a small number of basis vectors.

## 1. Introduction

The challenge of continually improving the technology for generating noninvasive medical images of internal anatomy is an area of great interest in the area of radiology. In particular, computed tomography (CT) is one of the most common modalities used for noninvasive medical diagnostic imaging. Cross-sectional CT images are reconstructed from projection data, which is generated by passing X-rays through the target object and measuring the resulting attenuation of those rays. For some noninvasive imaging modalities, the measurements made (i.e., the projection data) can be converted into samples of a Radon transform [1] in order to be reconstructed. In CT, dividing the measured photon counts by the incident photon counts and taking the negative logarithm yields the linear attenuation map of each projection, which, when noted at each projection angle, can be used to determine the Radon transform of the object of interest.

In the fields of accelerator physics, one expects that the relatively simple charged particle beam distributions can be reconstructed from a small number of projections. Tomographic imaging involves the reconstruction of an image from its projections. The reconstruction problem belongs to the class of inverse problems, and it is characterized by the fact that the information of interest is not directly available for measurement. If **f** denotes the unknown distribution and **b** the quantity measured by the imaging device, then the measurement system is written as **b** = **R** **f** for the Radon transform **R** [1]. The discrete problem, which is based on expanding **f** in a finite series of basis function, can be written as **b** = **A** **f**. The matrix **A**, typically large and sparse, is a discretization of the Radon transform. An approximate solution to this linear system could be computed by iterative methods, which only require matrix-vector products and hence do not alter the structure of **A**.

There are numerous approaches to reconstruct the shape of the target object from numerous angular projections. The iterative reconstruction algorithms are known to generate higher quality CT images than the filtered backprojection (FBP) algorithm. For example, the algebraic reconstruction technique (ART) [2] efficiently solves the inverse problem. Yet, although ART is effective, it still produces noise within a reconstructed image. To reduce the noise from reconstructed images using ART, the postprocessing is required. Unfortunately, it is possible to lose important data while removing the noise. Here, we propose a sparse-coding-based reconstruction method to overcome the drawbacks of iterative reconstruction approaches like ART and to minimize the influence of the deficiency of the projection measurements that are very sparse, while the recovery is slightly impacted from random noises. The recovery is achieved by finding an approximation vector with small *ℓ*_1_ and *ℓ*_2_ norm errors, which provides a good fit locally and globally. Our contribution in this paper is to solve the inverse problem using sparse coding by reducing both the random noise and the dependency on projection scans, which is less dependent on the number of angular projections than previous iterative reconstruction methods.

## 2. Our Proposed Approach

To reconstruct the original shape from projected measurements, we regard an original image as a discrete function *f*(*x*, *y*) defined on a domain *Ω* = {(*x*, *y*) ∈ ℝ^2^ : 0 ≤ *x* ≤ *n*,  0 ≤ *y* ≤ *m*} such that *f*(*x*, *y*) is constant on each cell *I*_*i*,*j*_ = [*i* − 1, *i*] × [*j* − 1, *j*] for *i* = 1,…, *n* and *j* = 1,…, *m*. We number the cells in lexicographical order so that *I*_(*i*−1)*m*+*j*_ = *I*_*i*,*j*_ for *i* = 1,…, *n* and *j* = 1,…, *m*. Let *f*_*i*_ be the constant in the *i*th cell. For each angle *θ*_*k*_ given in degree, *k* = 1,…, *q*, we project the image along parallel rays *p* with width *w* as follows:

(1) $$\begin{array}{c} b_{i}=\sum_{j=1}^{N}a_{ij}f_{j},\;i=1,\ldots ,P\;\;\left(N:=nm\right), \end{array}$$

where *P* is the total number of projections, and the coefficient *a*_*ij*_ is defined to be the length (or area) of the *i*th ray through the *j*th cell and *a*_*ij*_ = 0 if the ray does not touch the cell; that is, the value *b*_*i*_ is the ray sum measured along the *i*th ray and *a*_*ij*_ is the weighting factor representing the contribution of the *j*th cell to the ray sum. The tomography problem is then related to the reconstruction of an unknown image **f** = (*f*_1_,…, *f*_*N*_)^*T*^ from the observed vector **b** = (*b*_1_,…, *b*_*P*_)^*T*^ given as

(2) $$\begin{array}{c} Af=b, \end{array}$$

**Figure 1:**
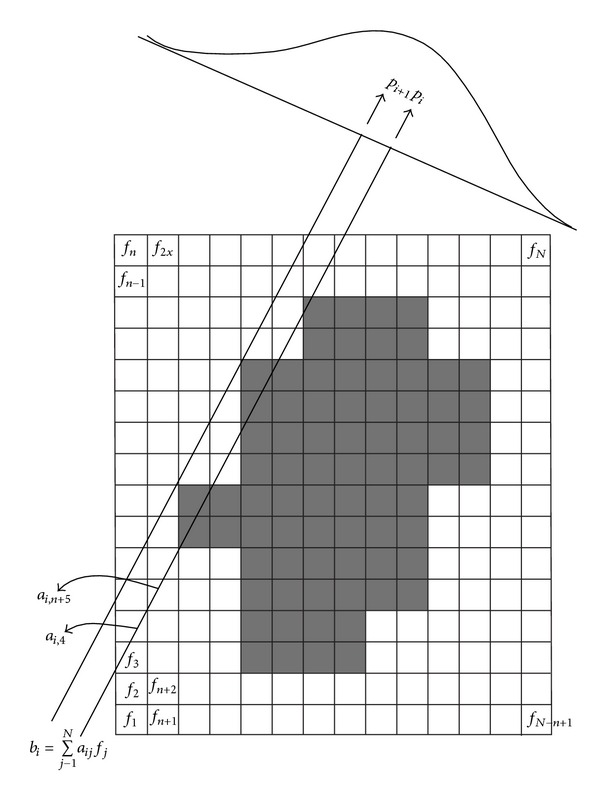
Our proposed CT image reconstruction method and its mathematical notations: *b*_*i*_ is the line integral of **f** along the ray *p*_*i*_, and *a*_*ij*_ is the length of the intersection of the ray *p*_*i*_ and the pixel *I*_*j*_.

Here, **A** = (*a*_*ij*_)_*i*=1,*j*=1_^*P*,  *N*^, and ·^*T*^ denotes the transpose of a vector/matrix. In general, *P* < *N*, and the problem is underdetermined and so is ill-posed.  Figure 1 shows our proposed CT image reconstruction approach and mathematical notations in (1) and (2).

This inverse problem emerges in areas of study involving 2D and 3D imaging such as medical imaging, geophysics, and material science [3–5]. To solve the inverse problem (2), many methods such as the least squares method, ART [6, 7], SARTs (simultaneous algebraic reconstruction techniques [8]), and SIRT (simultaneous iterative reconstruction techniques [9]) have been proposed and studied (see [10, 11] and references therein). We observe that almost all entries of **A** are zeros, and the vector **f** from CT images also has sparse pixel values. Based on this observation, we propose a method to solve the inverse problem by applying the compressive sampling (CS) technique. CS pertains to the recovery of **x** ∈ ℝ^*K*^ from rather a small amount of measurements **y** ∈ ℝ^*L*^ with *L* < *K*. Given an underdetermined systems

(3) $$\begin{array}{c} y=\Phi x, \end{array}$$

where Φ is an *L* × *K* matrix, CS enables us to perform exact recovery when **x** is sparse, and the matrix Φ satisfies the restricted isometry property [12, 13] (also see [14, 15]). The recovery performs through the so-called basis pursuit optimization:

(4) $$\begin{array}{c} \underset{x\in {\mathbb{R}}^{K}}{\arg\;\min}{||x||}_{1}\;\;\text{subject to}\;\;y=\Phi x. \end{array}$$

Using ||v||_*p*_ (*p* = 1,2), we denote the *ℓ*_*p*_ norm of a vector v = (*v*_1_,…, *v*_*K*_)^*T*^ ∈ ℝ^*K*^ defined by ||v||_*p*_ = (|*v*_1_|^*p*^ + ⋯+|*v*_*K*_|^*p*^)^1/*p*^. In the paper, we also consider the case where noise is added to the projected image, **f**, so that we can model such an imaging system as follows:

(5) $$\begin{array}{c} Af=b+\eta . \end{array}$$

In order to recover the sparse image from the inverse problem (5) while preserving intrinsic geometric structures such as edges, we try to find an image $\tilde{f}$ which provides a good fit to the original image **f** globally (small amount of ${||f-\tilde{f}||}_{2}$) and locally (small amount of ${||f-\tilde{f}||}_{1}$) as well. So, we propose the following elastic net optimization:

(6) $$\begin{array}{c} \tilde{f}=\underset{f\in {\mathbb{R}}^{N}}{\arg\;\min}\frac{1}{2}{||Af-b||}_{2}^{2}+\beta {||f||}_{2}^{2}+\gamma {||f||}_{1}. \end{array}$$

The model in (6) is a combination of the basis pursuit denoising (*β* = 0) and the ridge regression (*γ* = 0). In (6), the first term measures the fitting, and the third regularization term is added to recover a sparse data where *γ* controls the trade-off between sparsity and reconstruction fidelity. The quadratic term of the regularization gives rise to the grouping effect of highly correlated variables and removes the limitation on the number of selected variables [16]. The basis pursuit denoising enables us to reconstruct **f** simply in the *ℓ*_1_ sense, making **b** close to **A** **f** in the *ℓ*_2_ sense. We solve the optimization (6) using the feature-sign search algorithm [17].

## 3. Experiments

**Figure 2:**
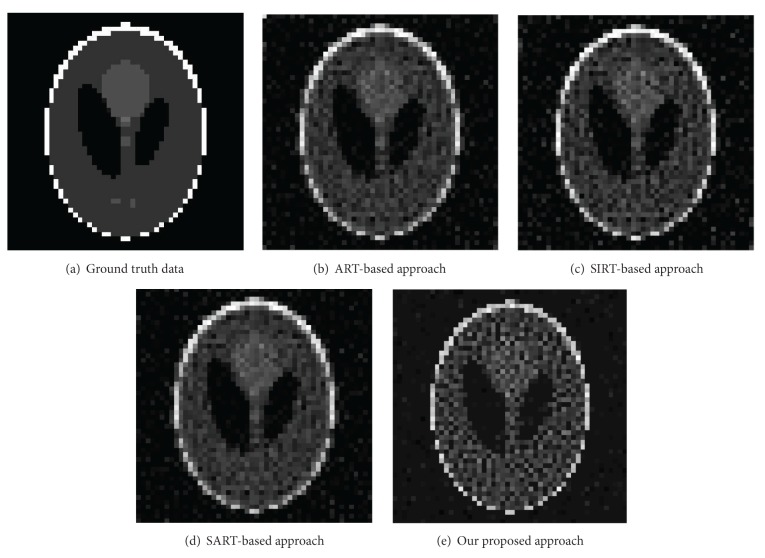
Comparison of image reconstruction from 2250 angular projections around the target object.

The schematic setup for sparse-coding-based CT image reconstruction is illustrated in this section. To efficiently show the robustness of our proposed approach, we have compared our sparse-coding-based image reconstruction method to other popular approaches, such as traditional ART, SIRT, and SART in Figure 2. For fair comparison, we reconstructed an image that has 50 × 50 resolution from 2250 angular projections around the target object. The previous approaches such as ART (Figure 2(b)), SIRT (Figure 2(c)), and SART (Figure 2(d)), require several iterations to robustly reconstruct the target object (100 iterations in our experiment because the reconstructed images have few improvements in resolution after that.) As shown in Figure 2, there is still a noise effect in the background, but our proposed sparse-coding-based approach (Figure 2(e)) dramatically reduces the noise in the background within a reconstructed image.

**Figure 3:**
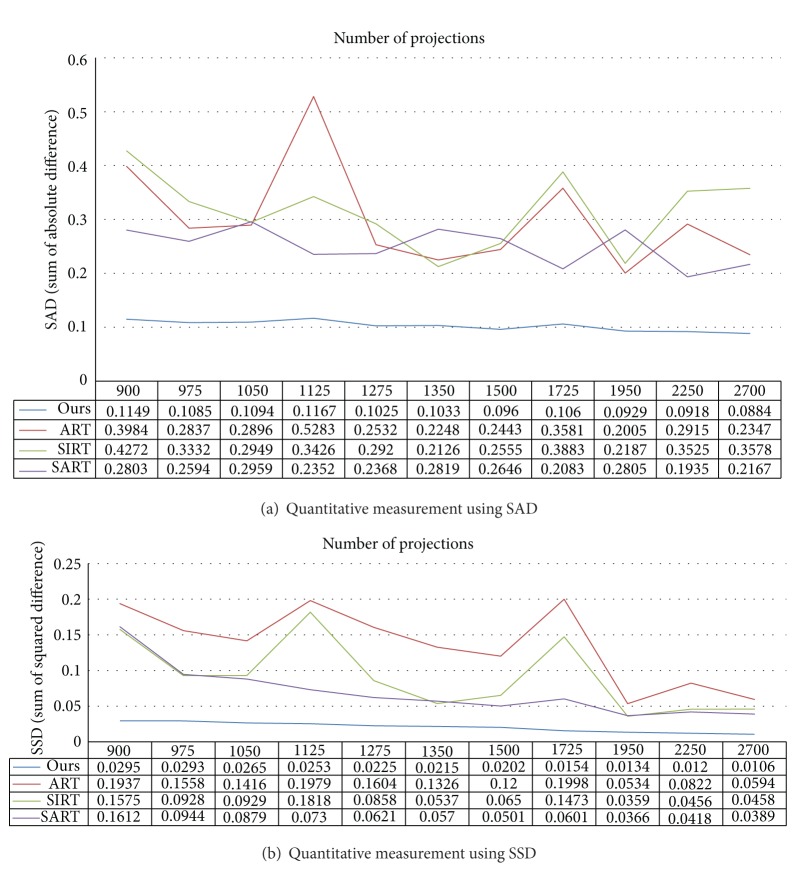
Quantitative comparison of previous approaches and our proposed approach using SAD and SSA by changing the number of angular projections from 900 to 2700.

Image reconstruction using rotated angular projection methodologies is very dependent on the number of projections. To show the robustness of the sparse-coding-based image reconstruction methodology, we have quantitatively compared our approach to previous approaches by changing the number of the projections. For this, we evaluated the *ℓ*_1_-norm-based similarity measure called SAD (sum of absolute difference) and *ℓ*_2_-norm based-similarity measure called SSD (sum of squared difference) between reconstructed images and the original image by changing the number of projections. In Figure 3, we show how we robustly reconstruct the original image by calculating the SAD and SSD from the randomly selected projections from 900 to 2700 around the ground truth data between the reconstructed images using ART, SIRT, SART, and our approach and ground truth data. Figure 3 demonstrates that our proposed approach can reconstruct the original shape of the target object while reducing the effect of the number of projections. SAD-based error comparison as shown in Figure 3(a) provides that our proposed reconstruction method is less dependent on the measurement (*b*) than other previous approaches, and SSD-based comparison in Figure 3(b) shows that our proposed approach is very efficient to solve the inverse linear problem to recover the original shape of the target object.

## 4. Discussion

In this paper, we propose a novel reconstruction approach from the projected measurement of the radiation around the target object. This nonparametric approach to the reconstruction of medical CT images using sparse coding shows robustness in the reconstruction of the original shape without prior information of the target object. Sparse coding is a state-of-the-art technique used to reconstruct a sparse data set from a small number of linear measurements. We propose a reconstruction method based on sparse coding, motivated by the fact that medical images are often represented in terms of only a few data pixel values in the spatial domain. Our proposed image reconstruction methodology is less dependent on the number of projections collected around the target object, so the original shape can be recovered with only a few measurements. Based on our experimental evaluation, we argue that the proposed sparse-coding-based CT image reconstruction scheme provides better *ℓ*_1_-norm and *ℓ*_2_-norm error results than previous approaches.
